# New insights into APCVD grown monolayer MoS_2_ using time-domain terahertz spectroscopy

**DOI:** 10.1038/s41598-023-31102-z

**Published:** 2023-03-13

**Authors:** Saloni Sharma, Pooja Chauhan, Shreeya Rane, Utkarsh Raj, Shubhda Srivastava, Z. A. Ansari, Dibakar Roy Chowdhury, Bipin Kumar Gupta

**Affiliations:** 1 Photonic Materials Metrology Sub Division, Advanced Materials and Device Metrology Division, CSIR-National Physical Laboratory, Dr. K. S. Krishnan Road, New Delhi, 110012 India; 2 Academy of Scientific and Innovative Research (AcSIR), Ghaziabad, 201002 India; 3 Mahindra University, Bahadurpally, Hyderabad, Telangana 500043 India; 4 Centre for Interdisciplinary Research in Basic Sciences, Jamia Millia Islamia, Jamia Nagar, New Delhi, 110025 India

**Keywords:** Applied physics, Condensed-matter physics, Optical physics, Quantum physics

## Abstract

In modern era, wireless communications at ultrafast speed are need of the hour and search for its solution through cutting edge sciences is a new perspective. To address this issue, the data rates in order of terabits per second (TBPS) could be a key step for the realization of emerging sixth generation (6G) networks utilizing terahertz (THz) frequency regime. In this context, new class of transition metal dichalcogenides (TMDs) have been introduced as potential candidates for future generation wireless THz technology. Herein, a strategy has been adopted to synthesize high-quality monolayer of molybdenum di-sulfide (MoS_2_) using indigenously developed atmospheric pressure chemical vapor deposition (APCVD) set-up. Further, the time-domain transmission and sheet conductivity were studied as well as a plausible mechanism of terahertz response for monolayer MoS_2_ has been proposed and compared with bulk MoS_2_. Hence, the obtained results set a stepping stone to employ the monolayer MoS_2_ as potential quantum materials benefitting the next generation terahertz communication devices.

## Introduction

The vision of wireless connectivity (Sixth generation connectivity, 6G) is drawing more and more attention due to its implications for the upcoming era of super smart society, artificial intelligence, virtual reality, smart wearable, autonomous vehicles, wireless local area networks (WLANs), high speed train (HST) communications and many more^1–3^. Idea behind 6G is to achieve the target of Tbps data transmissions with eco-friendly and green connectivity for the betterment of life quality for upcoming generation. As higher data traffic rate is mandatory in 6G wireless connectivity, introduction of underutilized terahertz (THz) frequency band is an important step^4,5^ by widening the operational band to THz frequency range and hence, doors for higher data rate and better spectral efficiency can be opened up. Typically, THz domain signifies the frequency band between microwave and optical frequencies as its spectral range lies between 100 GHz and 10 THz. This frequency range carries tremendous potential for deployment of novel futuristic technologies based on photonics as well as electronics. For real life application of the wireless communication system, suitable materials need to be utilized for related components which should be designed and assembled in efficient manner and compact in nature. Therefore, in search of desired THz materials, scientific community is now looking at newly emergent 2D materials that can replace existing bulk materials^6^. The atomically thin layered materials are considered exceptionally different due to their novel physical, chemical and electronic properties such as thermal stability, higher mobility, greater conductivity, bandgap tuneability, broadband optical response, and tuneable THz properties^7^. Thus, these materials exhibit great potential in dynamically controlling THz responsivity along with THz wave propagation^7^. Specific category of 2D materials i.e. transition metal diachalcogenides (TMDs) have captured significant attention because of stabilized carrier mobility and higher modulation efficiency. In this series, monolayer semiconducting TMDs with low dielectric screening reveal strongly bound excitons^8^. By using proper optical excitation, the excitonic properties of TMDs can be tuned in ultrafast reaction time of photoluminescence (PL)^9^. It is well established that the thinning down a bulk TMDs to monolayer or few layers, shifts the band gap from indirect to direct band gap^10^. Among all such TMDs, MoS_2_ could be the most suitable and promising candidate for THz applications because of its excellent electronic and photonic properties at room temperatures^11–14^_._ There are several methods to grow monolayer MoS_2,_ out of which, the atmospheric pressure CVD (APCVD) approach is strongly favoured due to the most consistent, high quality, large production and cost-effective along with vacuum-free and continuous in-line processes.

In present investigations, highly reproducible triangular shaped monolayer MoS_2_ have been synthesised using indigenously developed low cost APCVD set-up at “***CSIR-NPL***” with calibration of zone temperature, maintaining required temperature gradient throughout the quartz tube. The commercially available CVD set-up is equipped with two zone furnace system including expensive MFC (mass flow controller), whereas we developed a single zone CVD furnace set-up that is cost-effective and equipped with a calibrated high precision rotameter to control the inert gas atmosphere in quartz tube for synthesizing highly reproducible monolayer MoS_2_. The quality of as-synthesized triangular shaped monolayer MoS_2_ were characterized and validated through several standard microscopic and spectroscopic techniques. The major focus of the present work is to explore the terahertz characteristics of monolayer MoS_2_ using time-domain terahertz spectroscopy and comparative study with bulk MoS_2_ which is not reported so far. Additionally, the plausible mechanism of terahertz characteristics associated with monolayer MoS_2_ has also been advocated in details to realize its potential usage in emerging next-generation terahertz communication devices as compared to bulk MoS_2_.

## Results and discussion

**Figure 1:**
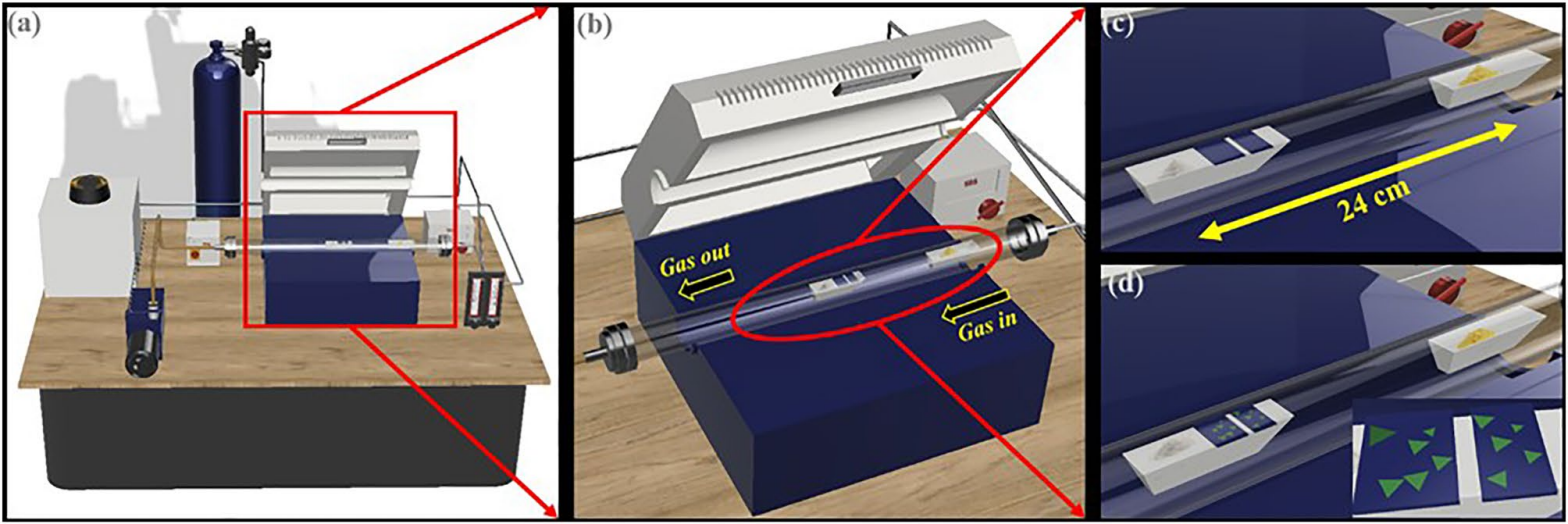
Pictorial representations of (**a**) Indigenously laboratory developed APCVD setup for synthesis of monolayer MoS_2_. (**b**) Horizontal split furnace and quartz tube. (**c**) Arrangement of precursors and substrate inside the furnace at constant heating zone before growth and (**d**) after growth.

Monolayer MoS_2_ was successfully synthesized on SiO_2_/Si substrates using single step APCVD growth technique using indigenously developed set-up at “***CSIR-NPL***”, New Delhi, India as shown in Fig. 1. Prior to the APCVD for growth of monolayer MoS_2_, all raw materials (MoO_3_ and S powder) utilized in the process were examined by XRD for purity and phase identification as shown in Fig. S1a,b. The quality growth, desired shape and size of monolayer MoS_2_ is dependent upon several important parameters such as growth time, growth temperature, heating rate, amount of gas flow and distance between the precursors^15^. The reaction of vapor phase includes two step process, first is formation of intermediate phase of MoO_3-x_ during which sulfurization of oxide takes place as shown in Eq. (1), followed by further saturation of sulfurization to complete the proper substitution of sulfur in place of unsaturated oxygen atoms formed during the first intermediate reaction^16,17^.

1 $$2{\text{MoO}}_{3} + x{\text{S}} \to 2{\text{MoO}}_{3 - x} + x{\text{SO}}_{2}$$

2 $$2{\text{MoO}}_{3 - x} + \left( {7 - x} \right){\text{S}} \to 2{\text{MoS}}_{2} + \left( {3 - x} \right){\text{SO}}_{2 }$$

The following equation is obtained after combining above Eqs. (1) and (2):

3 $$2{\text{MoO}}_{3} + 7{\text{S}} \to 2{\text{MoS}}_{2} + 3{\text{SO}}_{2}$$

**Figure 2:**
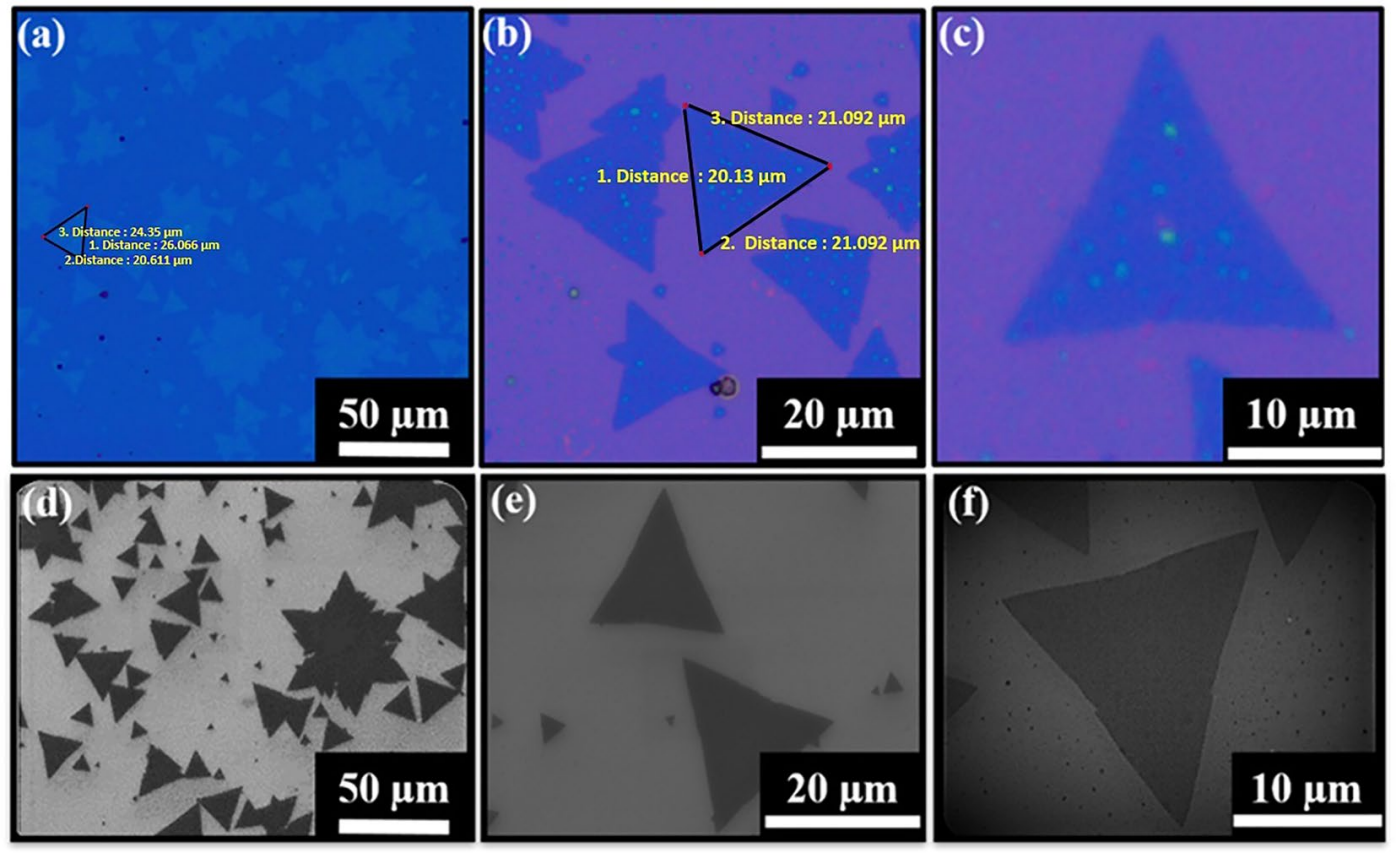
Optical and FESEM  Characterizations. (**a–c**) Optical images. (**d–f**) FESEM images at different magnifications of APCVD grown monolayer MoS_2_ over a 300 nm thick Si/SiO_2_ substrate, scale bar is mentioned.

**Figure 3:**
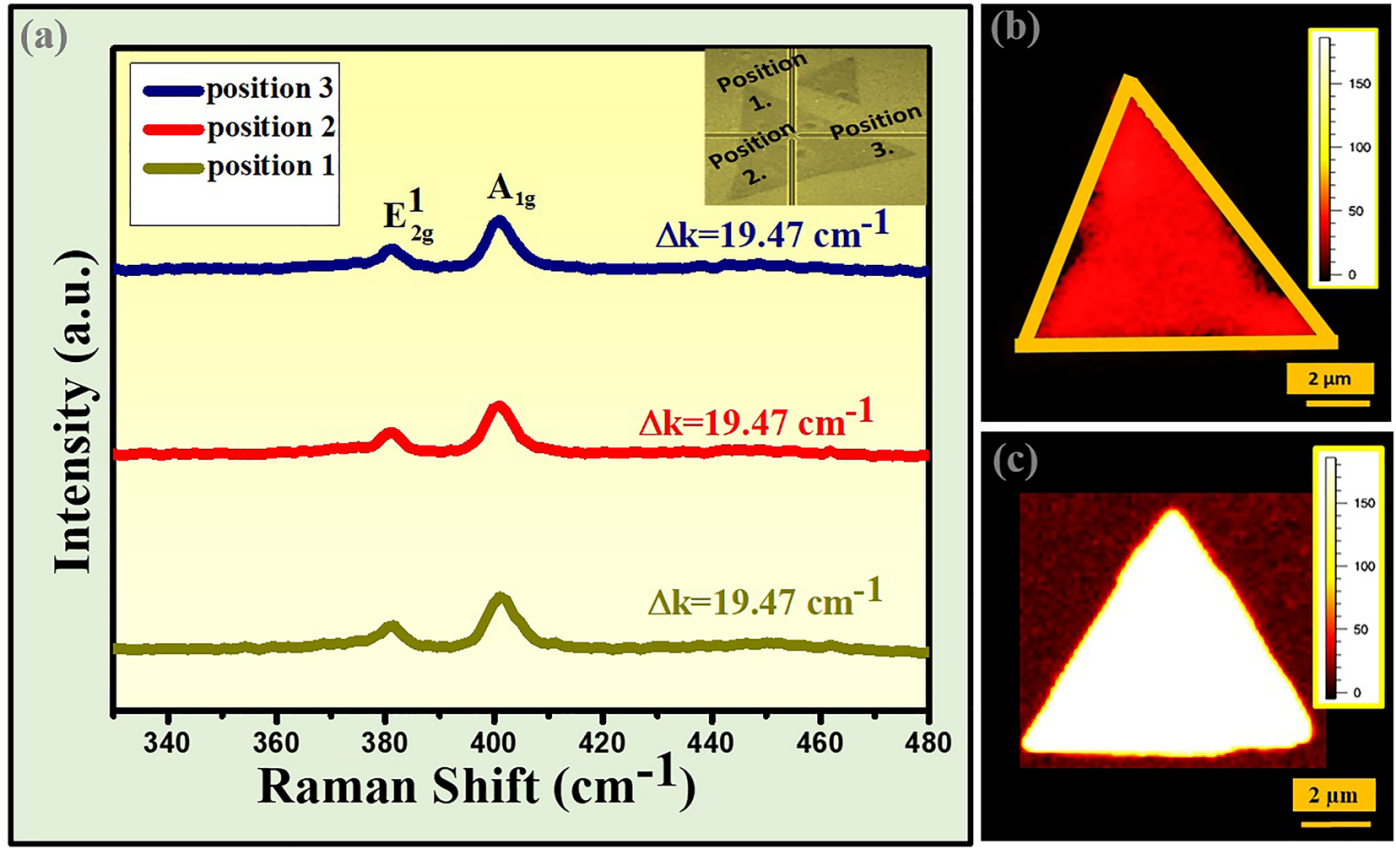
Raman spectra and Raman mapping. (**a**) Raman spectra of triangular shaped monolayer MoS_2_ on 300 nm thick SiO_2_/Si substrate at three of its corners represented with their frequency difference of in and out of plane vibration modes. Raman mapping of triangular shaped monolayer MoS_2_with respect to vibrational peak intensity. (**b**) In plane E^1^_2g_. (**c**) Out of plane A_1g,_ scale bar is mentioned.

In general, a complete sulfurization is essential for the growth of monolayer MoS_2_, while incomplete sulfurization is responsible for the growth of other secondary oxide phase (MoO_2_/MoO_3_) of molybdenum. To avoid the incomplete sulfurization, the rate of sulfurization can be controlled by changing the position of the sulfur boat to the center of heating zone, where the substrate was placed. Furthermore, by reducing proportion amount of sulfur to precursor (molybdenum oxide; MoO_3_) the growth of high-quality triangular shaped monolayer MoS_2_ can be controlled. Although, to optimize the condition, several statistical runs of APCVD were performed by varying the weight ratio of sulfur to MoO_3_ and different morphologies as well as size of monolayer MoS_2_ were deposited. The optimization of particular triangular shape has been done as reported in our previous studies^15,18^. The customized growth condition of present investigation has been discussed in details in experimental section. Further, to validate the number of layers of MoS_2_ in terms of qualitative analysis, Raman spectroscopy was performed. Prior to Raman spectroscopy, the shape, size (lateral dimension) and thickness contrast of as-deposited MoS_2_ layer on SiO_2_/Si substrate were probed by optical and field emission scanning electron microscopy (FESEM) techniques as shown in Fig. 2. The optical micrographs of as-deposited MoS_2_ layer on SiO_2_/Si at different magnifications have been shown in Fig. 2a–c. The morphologies of APCVD deposited MoS_2_ appear in triangular shape with maximum lateral size of 26.06 μm have been observed as shown in Fig. 2a and its contrast appears as atomically thin layer^19^. The maximum average lateral size is estimated to be 21.34 μm with number density of ~ 0.006/μm^2^ through FESEM as shown in Fig. 2d–f. The estimation of number density of monolayer MoS_2_ is described in details in the supporting note S2 and Fig. S2a,b (supporting information). The estimation of number density of monolayer MoS_2_ is based on selective area of FESEM image as shown in Fig. S2a. The statistical areal distribution of as grown monolayer MoS_2_ is shown in Fig. S2b. The lateral size of atomically thin triangular shape MoS_2_ obtained from FESEM is in agreement with lateral dimension obtained from optical micrographs as shown above. The same atomically thin triangular shape MoS_2_ sample has been used here from different selective areas for optical micrographs and FESEM. Raman spectroscopy has been performed to confirm the quality and number of layers of as deposited MoS_2_ by APCVD as shown in Fig. 3a. The Raman results exhibit the presence of two vibrational modes related to in-plane vibration of Mo and S atoms (E^1^_2g_) at 381.57 cm^−1^, and out-of-plane vibration of S atoms (A_1g_) at 401.04 cm^−1^, respectively. The frequency difference (Δk) between E^1^_2g_ and A_1g_ modes found to be 19.47 cm^−1^ at three different spatial positions of triangle selected randomly and the peak width estimated from full width half maxima (FWHM) of E^1^_2g_ mode which is found to be 4.76 cm^−1^ are in good agreement with the previously reported values of the APCVD-grown monolayer MoS_2_^18,20–25^. Furthermore, Raman mapping has been performed to reveal the uniformity of vibrational modes throughout the triangular surface of as deposited monolayer MoS_2_. The Raman mapping images of the triangular surface of as deposited monolayer MoS_2_ evidence the uniform spatial distribution of Raman intensity for E^1^_2g_ and A_1g_ vibration modes across the surface as shown in Fig. 3b,c. Thus, the Raman mapping confirms that the grown MoS_2_ layer, consisting of triangular shapes is monolayer in nature. Further, the absorption spectrum of monolayer MoS_2_ has been recorded for determination of optical transition as represented in Fig. S3. The UV–visible absorption spectrum and tauc plot of monolayer MoS_2_ have shown in Fig. S3a,b, respectively. The A excitonic peak (632 nm) emerges due to direct excitonic transition while B excitonic peak (612 nm) arises because of spin–orbit coupling as shown in Fig. S3a. Tauc equation has been employed to calculate the direct band gap of monolayer MoS_2_ which is 1.82 eV as shown in Fig. S3b.

4 $${\text{Tauc equation:}}\;\;\;\;\;\left( {\alpha h\upsilon } \right)^{\gamma } = A\left( {h\upsilon - E_{g} } \right)$$

where α: absorption coefficient, h: Planck’s constant, ν: frequency, A: proportionality constant, E_g_: band gap energy, γ: nature of electronic transition (for direct allowed transition, γ = 2)

**Figure 4:**
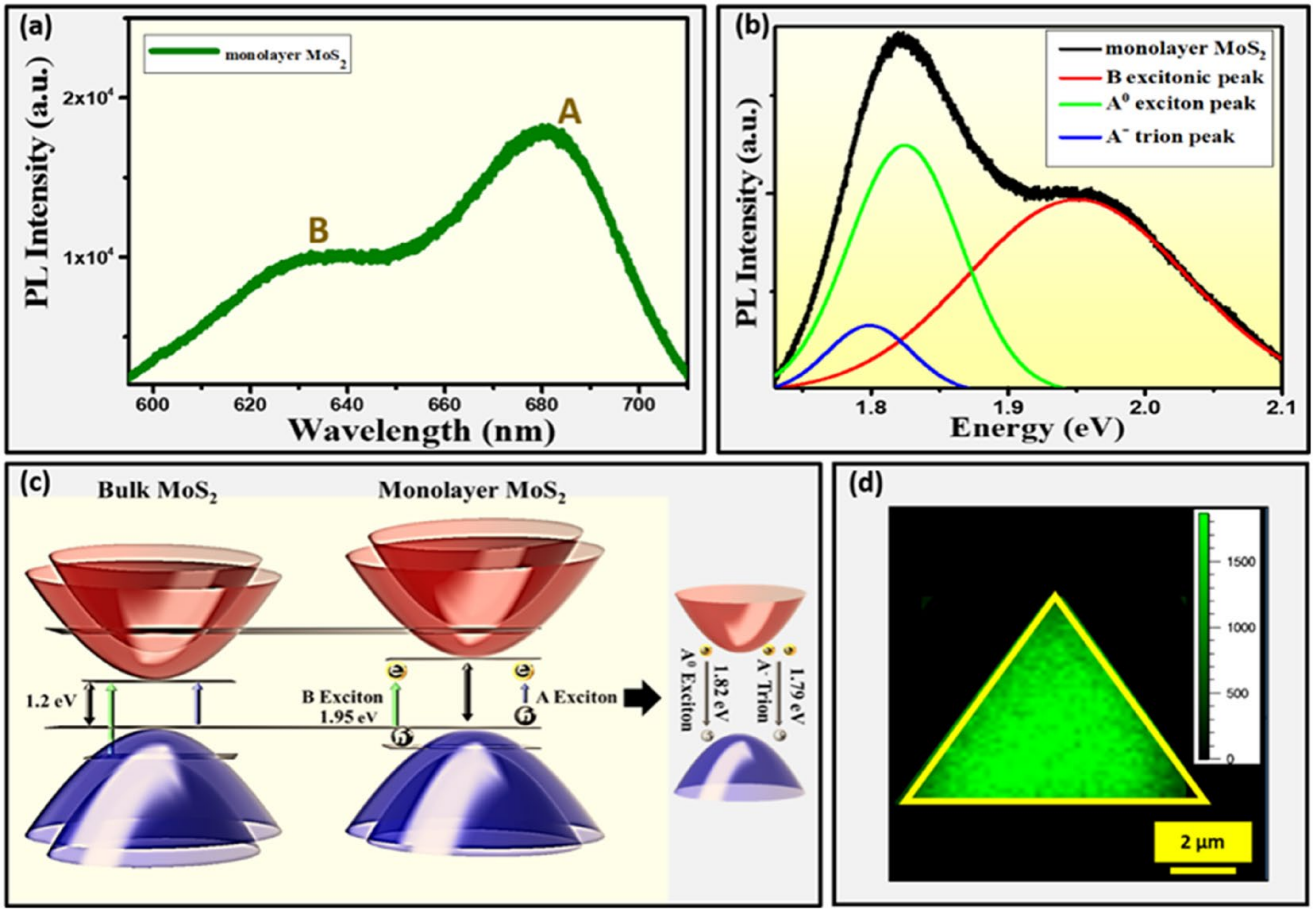
PL Characterization and Mechanism. (**a**) PL spectrum of monolayer MoS_2_ over 300 nm thick SiO_2_/Si substrate. (**b**) Deconvoluted PL spectrum of monolayer MoS_2_. (**c**) Proposed energy level diagram. (**d**) PL mapping of monolayer MoS_2_ with respect to A excitonic peak, scale bar is mentioned.

Furthermore, photoluminescence (PL) has been employed to analyse the quality of MoS_2_ monolayer at room temperature as shown in Fig. 4a. The PL spectra of triangular shaped MoS_2_ monolayer exhibits two distinct emission peaks upon excitation with diode laser of wavelength of 532 nm (2.33 eV). Among them, one of the strongest A excitonic emission peak is present in the range of 675 nm (1.83 eV) to 686 nm (1.80 eV) and centred at 680.86 nm (1.82 eV). While, another comparatively weak excitonic emission peak B exhibits in the range of 624 nm (1.98 eV) to 642 nm (1.93 eV), and centred at 633.82 nm (1.95 eV). The A and B excitonic emission peaks arise due to spin split direct transitions at K point of the Brillouin zone^26–29^. The PL emission intensity ratio of A to B excitonic peak was calculated to be 1.77 which again confirms the good quality of monolayer MoS_2_. The higher intensity of excitonic peak A than B implies the direct band transition in monolayer MoS_2_. Thus, the obtained PL result suggests that the quality of the MoS_2_ is monolayer in nature with direct band gap of 1.82 eV as compared to indirect bandgap of bulk MoS_2_ i.e.1.23 eV, which is also further supported by the above estimated band gap from absorption spectrum using Tauc plot and is in good agreement with several recently published PL results^18,30^. Further, a deconvolution analysis of the observed PL spectrum was carried out by fitting with three Gaussian peaks i.e. the neutral exciton (A^0^), negative trion (A^−^) and B exciton. The A^0^ and B exciton peaks are associated with the direct band gap transition at K point in the Brillouin zone, with energy split from the strong valance band spin orbit coupling as shown in Fig. 4b. The deconvolution result of PL spectrum exhibits A^o^ exciton energy of 1.82 eV^21^ while A^-^ trion energy has been 1.79 eV^18^. The PL intensity of A^o^ is observed to be much higher as compared to A^-^ trion, as shown in Fig. 4b. Moreover, for better understanding of PL emission process as well as dominance of A^o^ exciton as compared to A^-^ trion in as-synthesized monolayer MoS_2_, an energy level diagram has been proposed as shown in Fig. 4c. This energy level diagram also helps to understand the difference in PL process between bulk MoS_2_ indirect transition to monolayer direct transition. The theoretical prediction based on bulk MoS_2_ models estimates the indirect bandgap of bulk MoS_2_ as ~ 1.23 eV^26^. However, in case of monolayer MoS_2,_ it is interesting to know that A excitonic peak originates due to the change of excitonic binding energy or the gap between the lowest conduction band (CB) and the highest valence band at the K point of the Brillouin zone. One remarkable feature could be realized that when the layer thickness decreases from even few layers and bi-layer to monolayer, the PL intensity of A exciton is strongly enhanced, but that of the B exciton remains nearly constant as also experimentally observed by many research groups including our previous work related to quantification of number of layers of MoS_2_ using PL spectroscopy^19^. On the other hand, in case of bulk MoS_2_ the PL intensity of A and B excitons are almost similar^31,32^. While the presence of strong enhancement of the A exciton in monolayer MoS_2_ as compared to bulk can be implicit by the indirect to direct bandgap crossover as shown in Fig. 4a as well as in proposed energy level diagram in Fig. 4c, where the stark contrast of B exciton is showing a nearly constant intensity even when the thickness is changed from mono to bi-layer as reported in our previous studies too^19^. On the ground of experimental observations, the proposed energy level diagram represents the A and B excitonic transitions including the possibility of dominance of the presence of A^o^ exciton as compared to A^−^ trion defect level which is originated due to charge impurity present in as-synthesized monolayer MoS_2._ The lesser presence of A^−^ trion defect level exhibits the better crystal quality of as-synthesized monolayer MoS_2_. Furthermore, the dominance of A^o^ exciton interaction with conduction band electron of higher energy creates a strong charge-dynamic coupling which is strongly impactful to generate terahertz characteristics as further realized in THz-TDS results. Moreover, the spatial PL intensity distribution and uniformity of individual triangular shaped monolayer MoS_2_ throughout the surface was examined by using PL mapping technique where, 532 nm diode laser as a source of excitation with spot size of 1 μm was used as shown in Fig. 4d. The PL mapping result reveals that the PL intensity of the as–synthesized monolayer MoS_2_ is excellent with almost uniform throughout the triangular region^33^.

**Figure 5:**
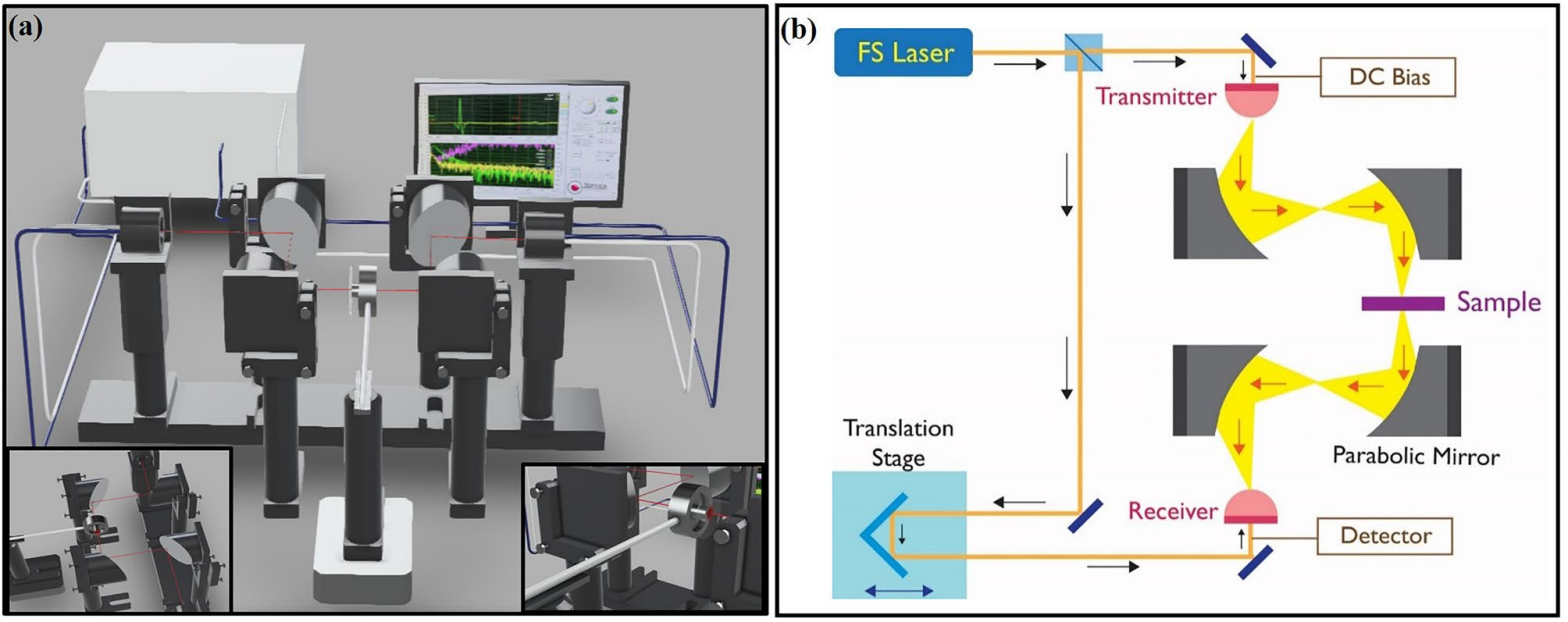
Time-domain terahertz spectroscopy set-up. (**a**) Pictorial view of Time-domain terahertz spectroscopy system and insets show the arrangement of parabolic mirrors and sample holder. (**b**) Schematic representation of THz-TDS mechanism.

Furthermore, the terahertz time-domain spectroscopy (THz-TDS) has been performed to explore the terahertz characteristics of as deposited monolayer MoS_2_ and bulk MoS_2_ on sapphire substrate by APCVD technique as shown in Fig. S4^34^. To satisfy the need of highly resistive substrate in order to nullify the conductive losses in substrate^35^ for THz-TDS measurements, the sapphire substrate has been chosen for growth of monolayer MoS_2_. The already optimized growth parameters have been used for growth on sapphire substrate as discussed in the experimental part. In order to validate the quality of as grown material on sapphire, optical microscopy and Raman spectroscopy have also been performed on sapphire substrates and shown in Figs. S5 and S6, respectively. A femtosecond (FS) laser in combination with photo conductive switch antennas are used for the generation and detection of terahertz pulses in order to perform THz-TDS on the samples. Briefly, THz-TDS technique employs a FS laser beam, which further splits into two parts. The actual set-up of THz-TDS as shown in Fig. 5a,b, depicts a schematic representation of THz-TDS mechanism. In THz-TDS set-up, the laser beam arrangements have been adapted in two different paths to generate and detect THz. In the first path, the split laser beam travels directly to terahertz emitter where it interacts with (photo conducting antenna) PCA to generate THz pulses, while in the other path, split beam reaches the detector in order to detect the generated THz pulse that passes through the sample (MoS_2_ on sapphire substrate in this study) (see, Fig. 5b). In such a way detected time-domain terahertz pulses are further converted to frequency domain spectra through Fast Fourier Transformations (FFT) resulting in complex frequency domain spectrum, as represented in the below equation:

5 $${\mathbf{E}}\left( {\mathbf{t}} \right)\to ^{{{\mathbf{FT}}}} \frac{1}{{\sqrt {2{{\varvec{\uppi}}}} }}\mathop \smallint \limits_{ - \infty }^{ + \infty } {\mathbf{E}}\left( {\mathbf{t}} \right){\mathbf{e}}^{{ - {\mathbf{i\omega t}}}} {\mathbf{dt}} = {\mathbf{E}}\left( {{\varvec{\upomega}}} \right)$$

Therefore, THz-TDS can provide information about both amplitude and phase. Further, with suitable referencing, such transmission measurements can provide frequency dependent information related to various optical parameters, such as, conductivity, refractive index, absorption coefficient etc. of the material under study. It is noticeable that the THz-TDS are performed at room temperature in dry atmosphere in order to negate the influence of humidity in atmosphere, hence capturing the genuine responses of MoS_2_ as closely as possible. The precision of the measurement of time-domain terahertz spectroscopy was calibrated in air as well as with known commercial sample for terahertz spectroscopy in terms of responsivity, transmission and sheet conductivity.

Further, Tinkham approximation has been used for analyzing the behaviors of ultrathin films of monolayer MoS_2_^36^. Therefore, frequency dependent conductivities are estimated using the following relations:

6 $${{\varvec{\upsigma}}}\left( {{\varvec{\upomega}}} \right) = \frac{{1 + {\mathbf{n}}}}{{{\mathbf{Z}}_{0} }}\left( {\frac{1}{{{\mathbf{T}}\left( {{\varvec{\upomega}}} \right)}} - 1} \right)$$

7 $${\mathbf{T}}\left( {{\varvec{\upomega}}} \right) = \frac{{{\mathbf{FFT}} [{\mathbf{E}}_{{{\mathbf{sample}}}} ({\upomega })]}}{{{\mathbf{FFT}}[{\mathbf{E}}_{{{\mathbf{substrate}}}} ({\upomega })]}}$$

here FFT: Fast Fourier transformation, T (${\upomega }$): Transmission, n_s_: refractive index of substrate, Z_0_: vacuum impedance (377 Ω), E_sample_(${\upomega }$): THz EF signal through sample, E_substrate_(${\upomega }$): THz EF signal through bare substrate

**Figure 6:**
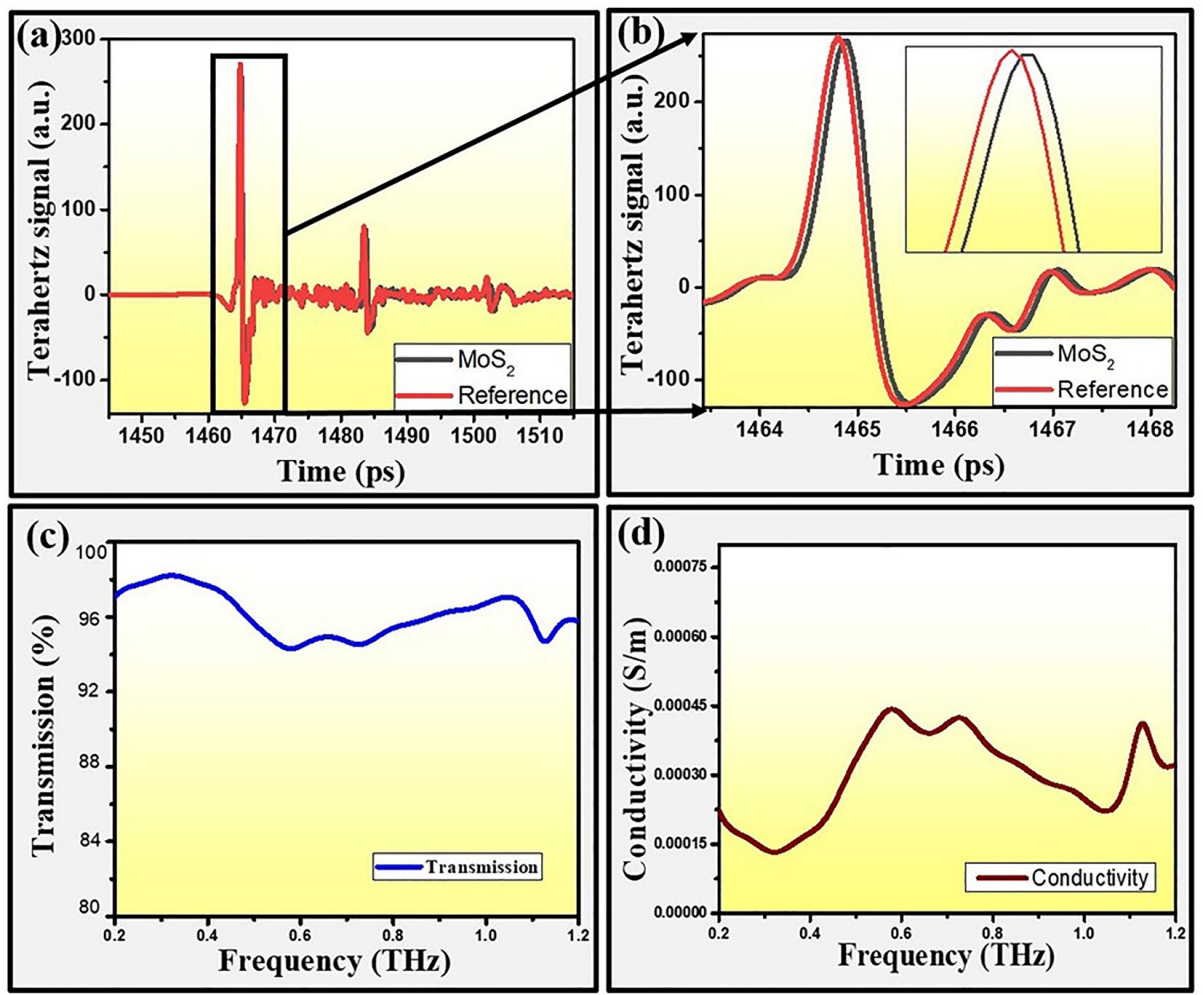
Time-domain terahertz spectroscopy. (**a**) THz signal transmitted through monolayer MoS_2_ on sapphire substrate. (**b**) Magnified view of transmitted THz pulse. (**c**) Transmitted THz amplitude through monolayer MoS_2_ grown on sapphire substrate. (**d**) Extracted sheet conductivity of monolayer MoS_2_ on sapphire substrate in THz frequency domain.

**Figure 7:**
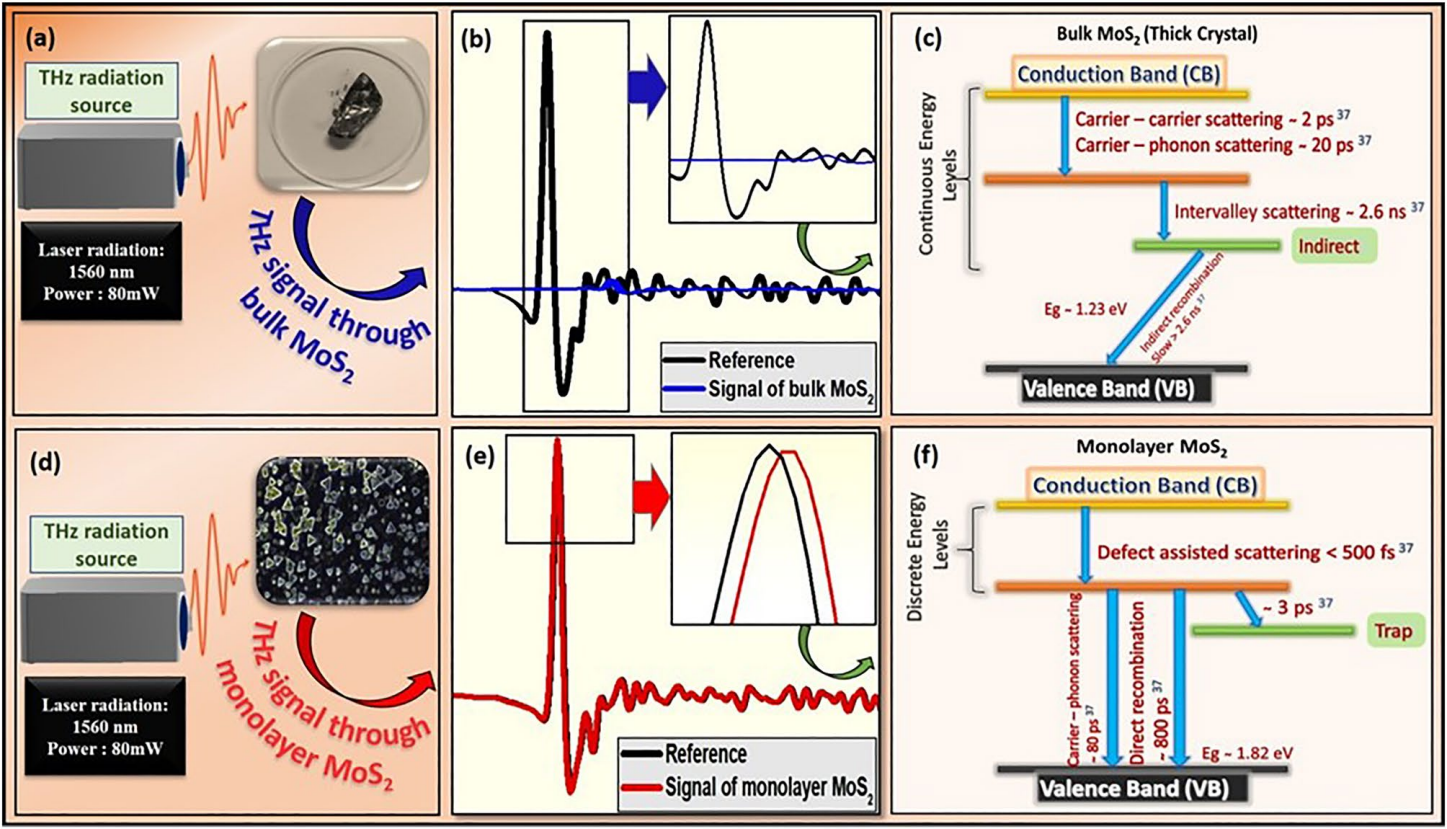
Comparative study of THz characteristics of bulk and monolayer MoS_2_. (**a**) THz signal incident on bulk MoS_2_. (**b**) THz signal transmitted through bulk MoS_2_. (**c**) Energy level diagram of bulk MoS_2_. (**d**) THz signal incident on monolayer MoS_2_. (**e**) THz signal transmitted through monolayer MoS_2_. (**f**) Energy level diagram of monolayer MoS_2_.

The observations for time-domain terahertz spectroscopy were carried out in denser areas of triangular shaped monolayers MoS_2_ (greater than terahertz spot size) which was confirmed through optical microscopy prior to measurement. Figure 6 depicts the terahertz signal transmitted through the monolayer MoS_2_. The transmitted terahertz pulse through the monolayer MoS_2_ has been first converted into frequency domain spectra using FFT and analyzed further. THz-TDS measurements of monolayer MoS_2_ show that monolayer MoS_2_ can modulate radiations as well as demonstrate certain responsivity in THz region as shown in Fig. 6a–d. The THz pulses transmitted through the monolayer MoS_2_ on sapphire substrate along with bare sapphire substrate^36^ are shown in Fig. 6a. However, Fig. 6b is the magnified view of pulses which are shown in Fig. 6a to depict the changes in transmitted pulses closely. Further, THz conductivities extracted using Tinkham relation are shown in Fig. 6d for 0.2–1.2 THz frequency range. Transmission spectrum through monolayer MoS_2_ demonstrates high transmission of almost 94–97% which is observed due to higher energy bandgap present in monolayer MoS_2_ as shown in Fig. 6c. The extracted sheet conductivity of APCVD grown monolayer MoS_2_ turn out to be around 1.3304 × 10^−4^ to 4.42 × 10^−4^ S/m for the similar THz frequency range. The unusual variations observed in conductivity may be explained on the basis of Ligand field theory. However, there is no direct evidence reported earlier for the same effects. Generally, this theory results from combining the principles laid out in molecular orbital theory and crystal field theory, which describes the loss of degeneracy of metal d-orbitals in transition metal complexes. It is interesting to observe that a small absorption of THz signal and blue shift were found due to the quantum confinement effect observed in monolayer MoS_2_. A strong spin–orbit coupling occurred between the conduction band electrons with A^0^ exciton direct band transition which is also supported by observed PL results as discussed earlier. Furthermore, for better clarity, a plausible mechanism has been proposed to explain the outstanding THz characteristics of atomic thin monolayer MoS_2_ as compared to bulk MoS_2_ crystal as shown in Fig. 7. Prior to perform the THz-TDS on bulk MoS_2_, the purity of crystal has been examined through XRD and Raman spectroscopy techniques as shown in Figs. S7 and S8, respectively. In present investigations, THz beam (with spot size 3–4 mm) is passed through monolayer MoS_2_ sample on substrate. Simultaneously, substrate is also measured prior to any deposition of monolayer MoS_2_ on it as shown in Fig. S9. Finally, THz signal passing through (MoS_2_ + substrate) is normalized with THz signal passed through substrate only. This way, we captured intrinsic true response of monolayer MoS_2_, which is devoid of any contribution from the substrate. The Fig. 7a–c represents the condition when terahertz signal interacts with bulk MoS_2_. The bulk MoS_2_ has lesser transmission of THz signal due to the continuous valance and conduction bands with indirect transition (band gap; Eg = 1.23 eV) which results in larger carrier-carrier scattering (2 ps)^37^, carrier-phonon scattering (20 ps)^37^ and intervalley scattering (2.6 ns)^37^. However, in case of monolayer MoS_2_, direct transition (band gap; Eg = 1.82 eV) with discreet energy levels are present because of quantum confinement effect, and hence defect assisted scattering (< 500 fs)^37^ and carrier-phonon scattering (80 ps) ^37^ are less dominating as shown in Fig. 7d–f. Therefore, transmission and sheet conductivity behave differently in case of thin monolayer MoS_2_ while it quenches significantly in case of bulk MoS_2_. Additionally, the terahertz signal is transmitted efficiently through monolayer MoS_2_ because resonance phenomenon occurs between incident and transmitted terahertz signals which can provide larger room for tunability for future THz applications^38^. This resonance phenomenon happens since the terahertz signal pulse width better synchronizes with monolayer MoS_2_ because of intraband direct transition time 350 fs reported^9^ earlier for monolayer MoS_2_ which is much smaller than the terahertz signal pulse width as shown in Fig. 7e. However, in case of bulk MoS_2_, the terahertz pulse does not synchronize with terahertz signal pulse width because of indirect band transition time is in the range of ps which is much larger than the terahertz signal pulse width in the range of fs. This could cause the major loss of terahertz signal in bulk MoS_2_, as a result the terahertz signal intensity in bulk MoS_2_ could significantly reduce as shown in proposed mechanism in Fig. 7b and major loss of terahertz signal, as also observed by other research groups for bulk MoS_2_^39^. We also performed two more measurements for monolayer MoS_2_ at different denser locations to ensure the successive repeatability of results as shown in Fig. S9. Thus, the time-domain spectroscopy results of monolayer MoS_2_ quantum material legitimate its potential use in wireless communication and also ignite us further to do more study for ultrafast, pump probe and 2D coherent spectroscopy to design the suitable next generation THz devices which will be our forth coming focus of study.

## Conclusions

A successful strategy has been proposed to synthesize high-quality monolayer MoS_2_ using indigenously developed APCVD set-up at CSIR-NPL, New Delhi, India. The optical and spectroscopic characterizations of monolayer MoS_2_ have been qualitatively validated through optical microscopy, Raman, UV–Vis and PL spectroscopic techniques which further quantified the deposited MoS_2_ as monolayer in nature. Moreover, the uniformity and spatial intensity distribution throughout the surface of monolayer MoS_2_ crystals was ensured through PL and Raman mapping and also the different in-plane and out-of-plane vibrations of Mo and S atoms were explored through mapping E^1^_2g_ at 381.57 cm^−1^ and A_1g_ at 401.04 cm^−1^, respectively. The morphologies and average lateral sizes of ~ 21.34 µm of triangular shaped monolayer MoS_2_ was confirmed through FESEM. Moreover, THz-TDS studies reveal conductivities in the range of 1.3304 × 10^–4 ^– 4.42 × 10^–4^ S/m for monolayer MoS_2_ within the frequency range of 0.2–1.2 THz frequency domain. The unexplored terahertz characteristics of monolayer MoS_2_ was explained in details and successfully proposed a plausible mechanism to explore the optical absorption introduced by electron-photon interaction in monolayer MoS_2_ including the role of exciton carrier dynamics of monolayer MoS_2_ due to quantum confinement effect and supported the argument through observed PL and terahertz characteristics. Thus, the obtained terahertz optoelectronic characteristics show potential of monolayer MoS_2_ as an impactful quantum material as compared to bulk MoS_2_ to participate in the development of next generation compact terahertz communication devices.

## Experimental

An indigenously laboratory developed APCVD set was used for the synthesis of monolayer MoS_**2**_ as shown in Fig. 1. The APCVD growth of monolayer MoS_**2**_ was optimized by varying the growth parameters such as gas flow, amount of precursors, distance between ceramic boats and heating rate etc.^40^. This process involves the evaporation of both metal oxide and chalcogenide precursors at different growth temperatures in horizontal quartz tube furnace. Several statistical runs of APCVD have been performed to optimize the condition on the basis of above mentioned parameters. The optimum condition was achieved with 15 mg of MoO_**3**_ (99.9%, Sigma Aldrich) and 100 mg of Sulfur (S) (CDH Delhi) in powder forms in ceramic boats (length 9 cm each) and placed in heating zone of furnace (24 cm distant from each other) in a quartz tube (specifications: 12.27 cm length, 6 cm inner diameter, 6.5 cm outer diameter) in horizontal split furnace. Further, cleaned SiO_2_/Si substrates were kept facing down on ceramic boat inside the furnace. Before heating the tube, it was evacuated with rotary pump for vacuum pressure of 4.2 torr to avoid any contamination of Oxygen during the growth. Further, the tube was purged with 480 sccm of Argon (Ar) gas for removal of different kinds of absorbed contaminants in tube such as moisture, dust etc.^16^. This inert environment of tube avoids any reaction with foreign particle impurities during the growth process. Heating ramp was set up for 30 min and was completed in two sequential steps^21,40,41^. In first step, gas flow was maintained same as before (480 sccm) up to 300 °C with a ramping rate of 14 °C/min. In second step, the gas flow rate was reduced to 120 sccm and ramping rate was allowed to increase up to 23 °C/min. Then, the vapor phase reaction started and growth was performed for 5 minutes^41^ at 655 °C. After growth, the furnace was allowed to cool naturally. Flow of inert gas was maintained inside the tube until the furnace cools down to 100 °C^16,34^. After the successful deposition of the monolayer MoS_**2**_ on SiO_2_/Si substrate, the substrate color was observed different from its body color prior to perform APCVD process. After successful deposition, the monolayer MoS_**2**_ was analyzed under optical microscope and other characterization techniques to validate its monolayer nature and quality. The optimum conditions were also examined through several sequential statistical runs and ultimately developed the strategy to grow highly reproducible monolayer MoS_**2**_ using indigenously developed APCVD.

### Characterization tools

To check the quality and purity of precursors prior to monolayer growth process using APCVD method, X-ray diffraction (XRD) has been performed for commercially purchased MoO_3_powder, S powder and MoS_2_ crystal through Rigaku made Mini Flex II X-ray diffractometer with Cu Kα_1_ (λ = 1.5418Å) radiation source. In order to visualize the synthesized monolayer MoS_2_ over the SiO_2_/Si substrate (GMS-India), optical microscopy is carried out. The LEXT 3D measuring laser microscope OLS5100 having the objective lenses MPLAPON50XLEXT, MPLAPON100XLEXT with NA and working distance of 0.95 and 0.35 mm, respectively is utilized in the bright field mode. Field emission scanning electron microscopy (FESEM) was used to investigate the surface morphology with Carl ZEISSSUPRA40 VP. Further, the Raman spectroscopy of monolayer MoS_2_ and bulk MoS_2_ crystal was performed using Renishaw inViaTM confocal Raman microscope (magnification: 50x, 100x) source of excitation laser wavelength was selected 532 nm/2.33 eV energy in backscattering mode at room temperature. UV–visible absorption spectrum of the monolayer has been recorded using a UV–Vis spectrometer (AvaLight-DH-S-BAL) to observe the excitonic states. The complementary PL spectroscopy was carried out using same instrument of Raman with configurational change at room temperature. The Raman and PL mappings were also performed with same Renishaw inViaTM confocal equipment at different spatial mapping configurations. The terahertz characteristics of monolayer MoS_2_ was extensively examined with the help of THz- TDS using TOPTICA FemtoErb THz FD6.5 (specifications: laser radiation 1560 nm, repetition rate 100 MHz, laser power 80mW).

## Supplementary Information

Supplementary Information.

## Data Availability

The datasets used and/or analysed during the current study available from the corresponding author on reasonable request.
