# Supplementary material for: New insights into APCVD grown monolayer MoS2 using time-domain terahertz spectroscopy
Source: Sci Rep. 2023 Mar 13;13:4146. doi: 10.1038/s41598-023-31102-z (PMC10011412; doi:10.1038/s41598-023-31102-z)
Supplement: Supplementary file 1 — Supplementary Information. [file 41598_2023_31102_MOESM1_ESM.docx]

**Supporting Information**

**New Insights into APCVD Grown Monolayer MoS_2_ using Time Domain Terahertz Spectroscopy**

Saloni Sharma,^1,2^ Pooja Chauhan,^1,2^ Shreeya Rane,^3^ Utkarsh Raj,^1^ Shubhda Srivastava,^1^ Z. A. Ansari,^4^ Dibakar Roy Chowdhury,^3^ and Bipin Kumar Gupta^1,2*^

^1^Photonic Materials Metrology Sub Division, Advanced Materials and Device Metrology Division, CSIR-National Physical Laboratory, Dr. K. S. Krishnan Road, New Delhi-110012, India, ^2^Academy of Scientific and Innovative Research (AcSIR), Ghaziabad-201002, India, ^3^Mahindra University, Bahadurpally, Hyderabad, Telangana 500043, India, ^4^Centre for Interdisciplinary Research in Basic Sciences, Jamia Millia Islamia, Jamia Nagar, New Delhi-110025, India

*Correspondence and requests for materials should be addressed to B.K.G. (email: [bipinbhu@yahoo.com](mailto:bipinbhu@yahoo.com))

The contents of supporting material are listed as follows:

**S1. XRD patterns of Molybdenum tri oxide (MoO_3_) and Sulfur (S) powder**

**S2. Calculation of number density of monolayer MoS_2_**

**S3. UV-Vis absorption spectra of monolayer MoS_2_**

**S4. Arrangement of sapphire substrates and powders in quartz tube inside the furnace**

**S5. Optical images of monolayer MoS_2_ on sapphire substrates**

**S6. Raman spectrum of monolayer MoS_2_ on sapphire substrate**

**S7. XRD patterns of bulk MoS_2_**

**S8. Raman spectrum of bulk MoS_2_**

**S9. Area selection for THz time-domain spectroscopic measurements of monolayer MoS_2_**

**Supporting Figures**

**
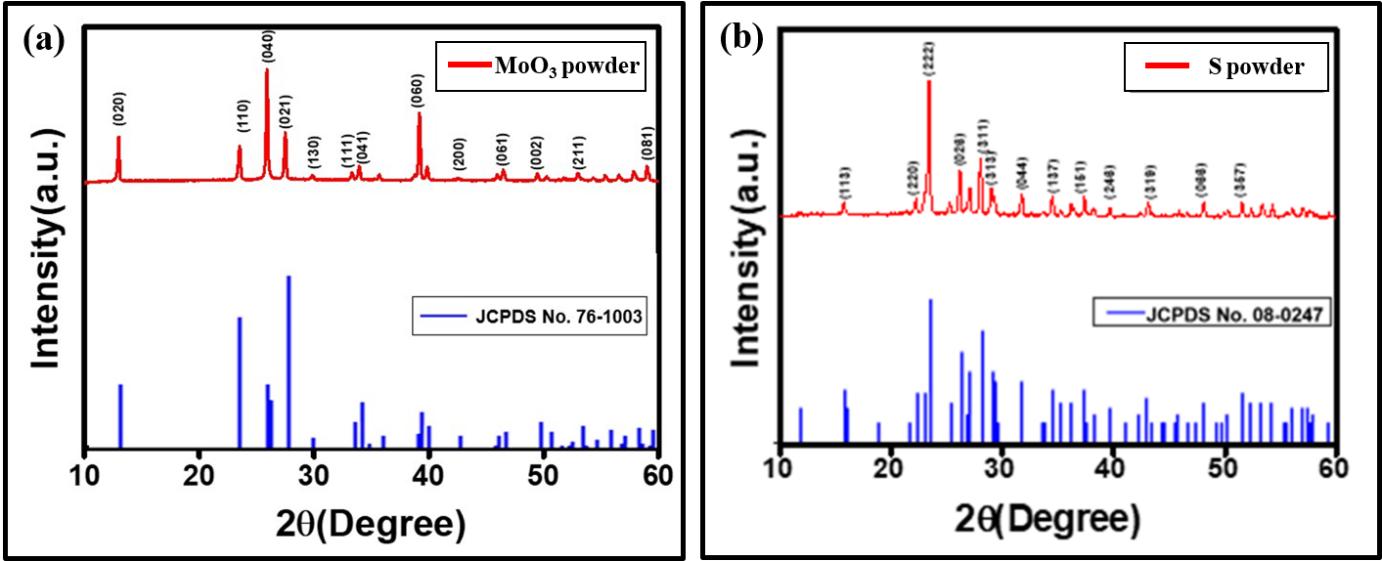
**

**Fig. S1.** XRD pattern representation of (a) MoO_3_ powder (b) S powder.

**
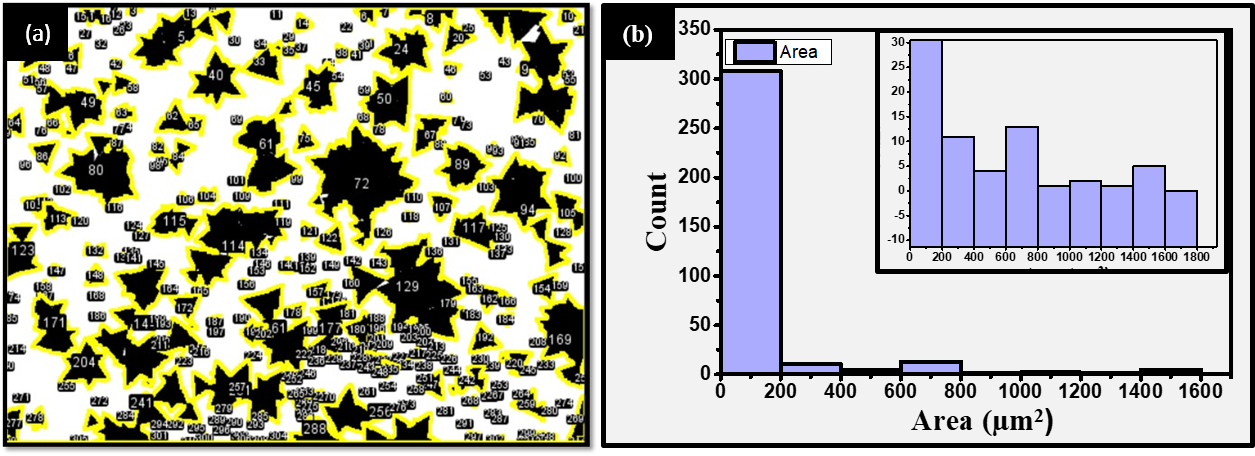
**

**Fig. S2.** (a) number counts of grown monolayer MoS_2_ (b) statistical histogram of counts with the areal range of monolayer MoS_2_ evaluated from ImageJ software.


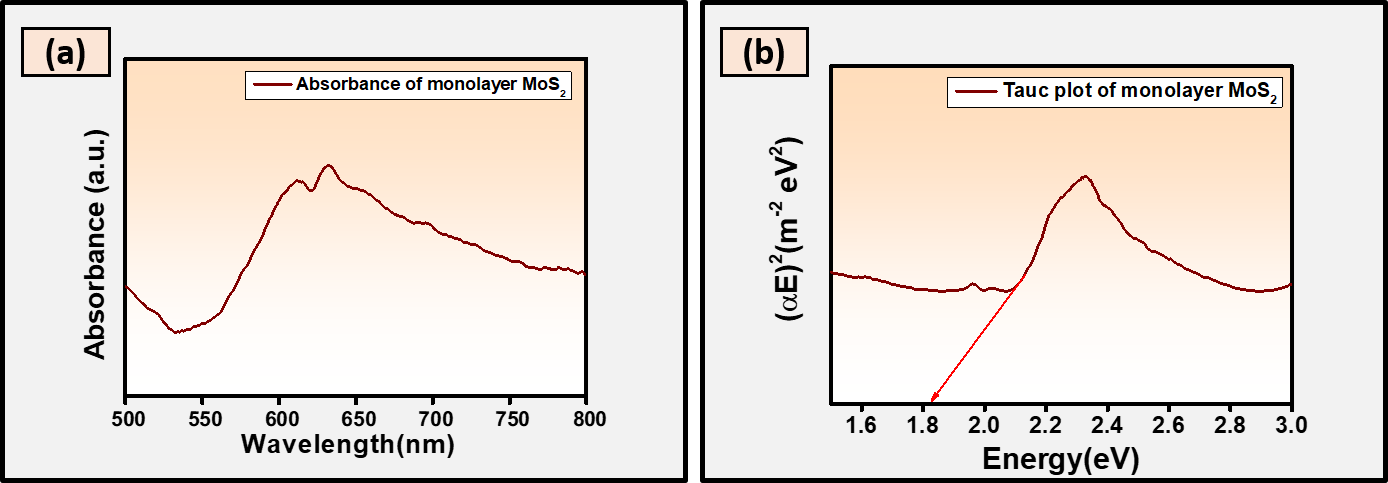


**Fig. S3.** (a) The absorption spectrum, (b) band gap/ Tauc plot of monolayer MoS_2_.

**
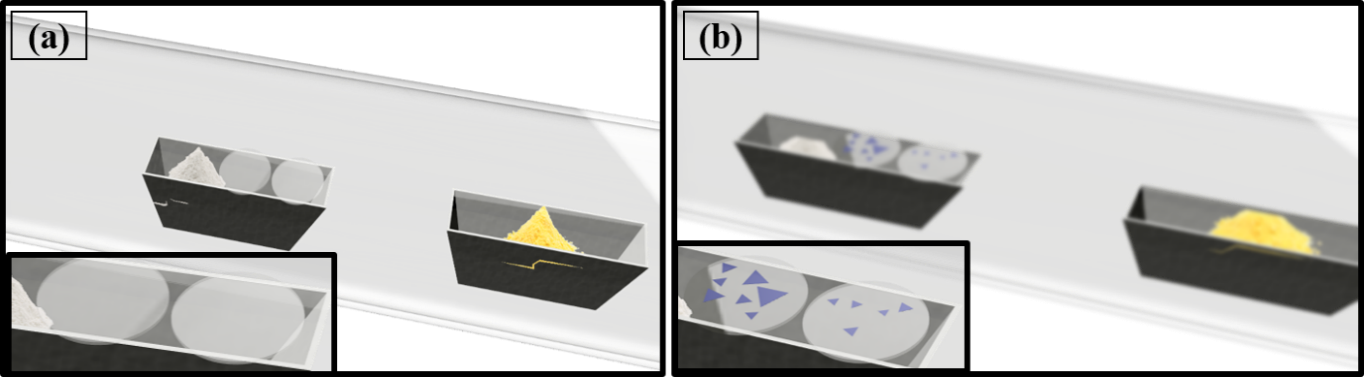
**

**Fig. S4**. Schematic view of sapphire substrates in quartz tube inside furnace (a) before sulfurization (b) during sulfurization.

**
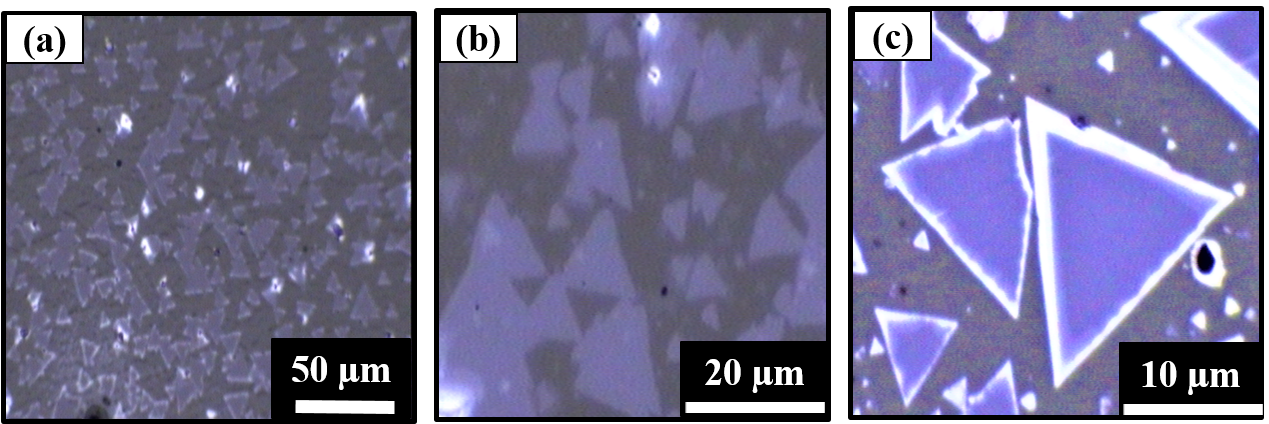
**

**Fig. S5.** Optical images of APCVD grown monolayer MoS_2_ on sapphire at different magnifications.

**
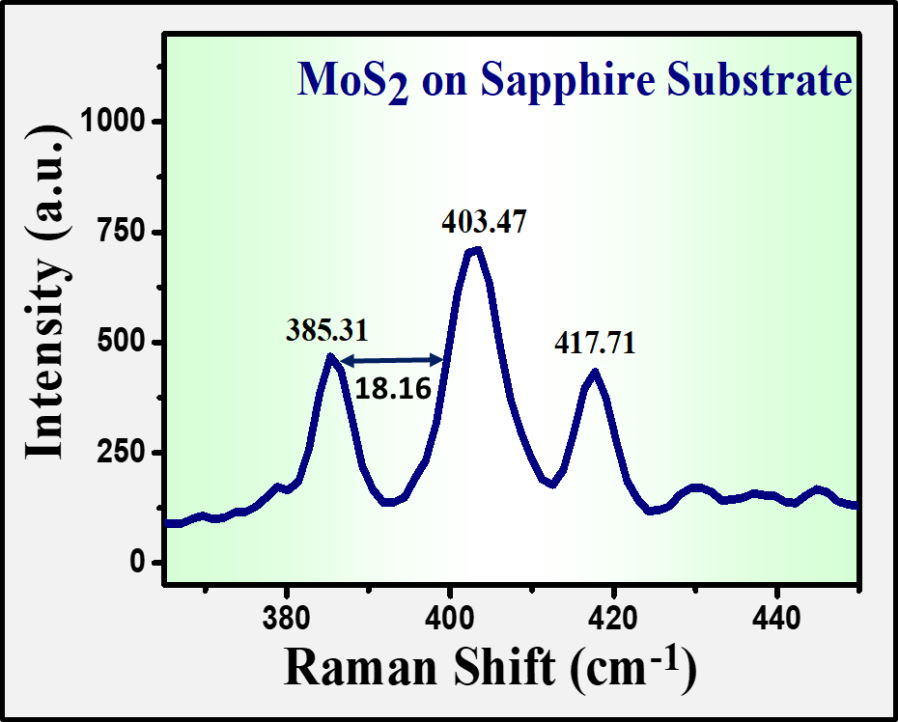
**

**Fig. S6.** Raman spectrum of monolayer MoS_2_ on sapphire substrate.


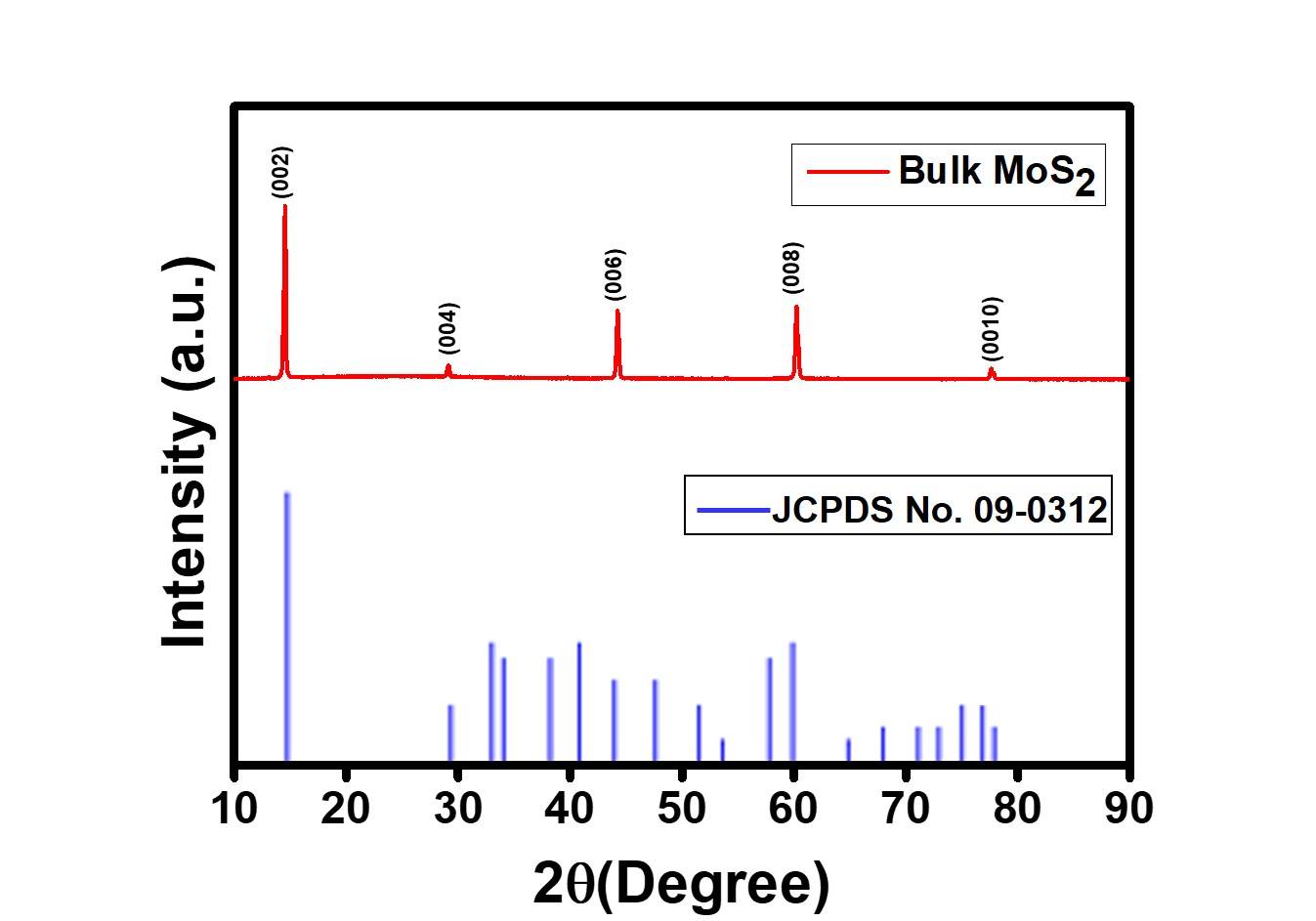


**Fig. S7:** XRD spectrum of bulk MoS_2_ crystal.

**
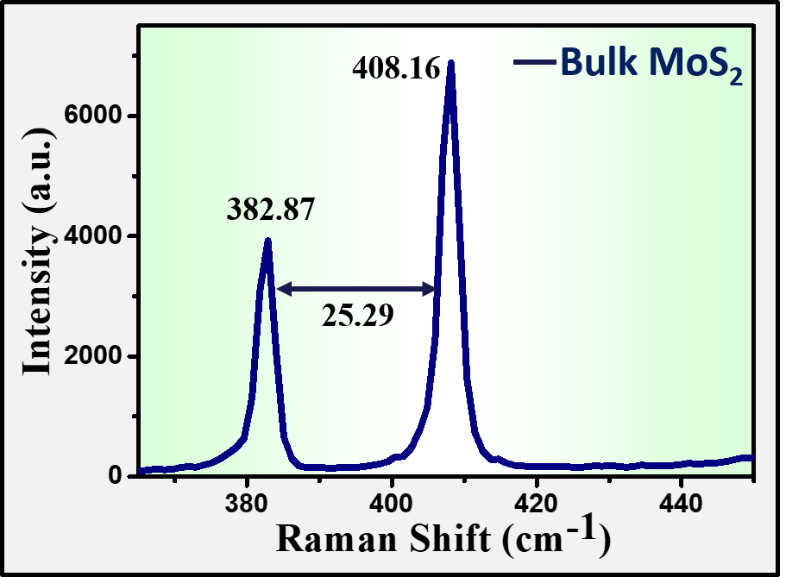
**

**Fig. S8:** Raman spectrum of bulk MoS_2_ crystal. Inset view is the optical image from where measurement has been taken.


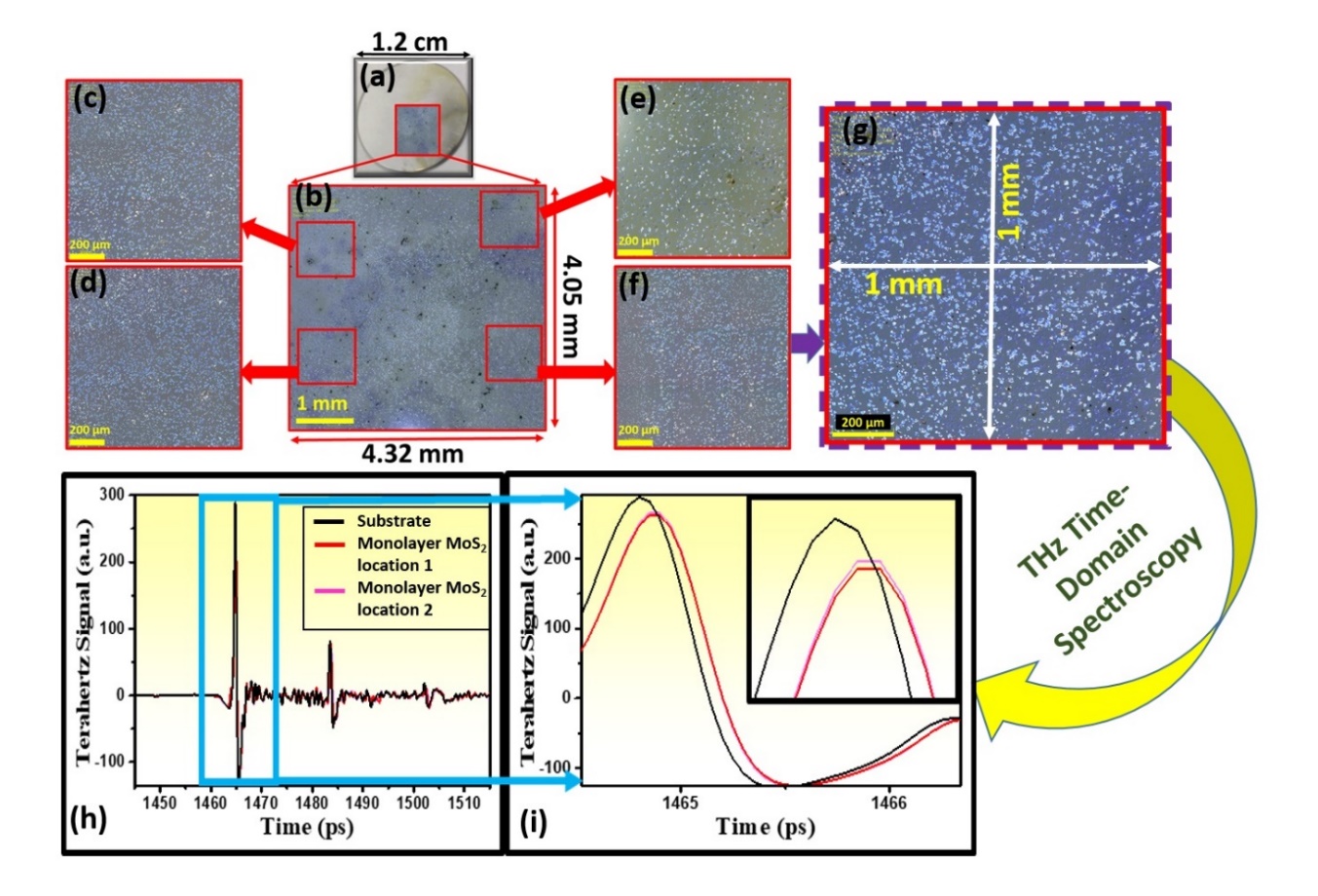


**Fig. S9:** (a) synthesized monolayer MoS_2_ on substrate. (b) magnified optical image of selected area for THz measurements. (c-f) selected magnified view of optically stitched images of figure (b), marked as rectangular regions (1mm x 1mm). (g) magnified view of optically stitched images of figure (f). (h) THz time-domain spectra for different location of monolayer MoS_2_ (red and pink plots) and bare substrate (black plot) are shown. (i) magnified view of figure (h).

**Supporting Notes**

**Supporting Note S1. XRD patterns of Molybdenum tri oxide (MoO_3_) and Sulfur (S) powder**

XRD has been carried out for both precursors to ensure about the quality of powders. Fig. S1a and S1b represents XRD pattern of MoO_3_ and S powder respectively. Peak positions of these XRD patterns of MoO_3_ and S has been indexed with JCPDS card 76-1003 and 08-0247 respectively.

**Supporting Note S2. Calculation of number density of monolayer MoS_2_**

SEM images are analyzed with the help of software ImageJ and further triangular number density was calculated. Fig. S2a represents the image of calculated number of synthesized monolayer MoS_2_ in chosen area using ImageJ software. Fig. S2b depicts the statistical histogram of covered monolayer MoS_2_ area versus the number of counts. It is clearly evident that maximum number of monolayer MoS_2_ is grown in areal range 0-200 µm^2^.

**Supporting Note S3. UV-Vis absorption spectra of monolayer MoS_2_**

Fig. S3a depicts the absorption spectrum with respect to wavelength of monolayer MoS_2_ and Fig. S3 b represents the tauc plot of monolayer MoS_2._

**Supporting Note S4. Arrangement of sapphire substrates and powders in quartz tube inside the furnace**

The optimized growth parameters were maintained same (as discussed in experimental section) for sapphire substrates. Two pretreated sapphire substrates were loaded on alumina boat inside reaction chamber of APCVD as shown in Fig. S4. Fig. S4a is pictorial representation of sapphire substrates and Fig. S4b is assumed depiction inside reaction chamber when sulfurization process was begun**^1-4^**.

**Supporting Note S5. Optical images of monolayer MoS_2_ on sapphire substrate**

The grown monolayer MoS_2_ on sapphire was analyzed under high resolution optical microscopy. The observed color contrast and shapes were observed at three different magnifications^1^.

**Supporting Note S6. Raman Spectrum of monolayer MoS_2_ on sapphire substrate**

The Raman spectrum of monolayer MoS_2_ has been recorded on sapphire substrate. Different peak values are identified at 385.31, 403.47 and 417.71 cm^-1^ respectively. The observed peak values of Raman spectrum have good agreement with previously reported values^5^. The frequency difference 18.16 cm^-1^ has been found between in and out of plane vibrations.

**Supporting Note S7. XRD patterns of bulk MoS_2_**

To check the purity of crystal, XRD spectroscopy has been carried out for bulk MoS_2_. Recorded XRD pattern of MoS_2_ crystal has been shown in Fig. S7. Peak positions of this pattern have been well indexed and have good agreement with JCPDS card no. 09-0312 and published literature^6^.

**Supporting Note S8. Raman spectrum of bulk MoS_2_**

Raman spectroscopy has been performed for bulk MoS_2_ to reassure about the purity of MoS_2_ crystal. The frequency difference between in and out of plane vibrational mode has been found 25.29 cm^-1^ which is clearly evident that crystal is in its pure state as per previously reported results^7^.

**Supporting Note S9. Area selection for THz time-domain measurements of monolayer MoS_2_**

To select the of higher density growth region (comparable to spot size) for THz time-domain measurements, the step-by-step procedure is discussed below:

Prior to measurements, an optical microscope was used to appropriately inspect the synthesized sample on sapphire of diameter 1.2 cm for high-density growth regions of monolayer MoS_2_, as shown in Fig. S9 (a). The rectangular geometry in Fig. S9 (a), depicts the region selected for higher density growth. Fig. S9 (b) shows the stitched optical image of the region selected [as shown Fig. S9(a) via red rectangular region] for measurements, which is 4.05 mm x 4.32 mm area. Furthermore, Fig. S9 (c-f) represent the segmented stitched optical magnified images to view the clear consistent growth of high-density of monolayer MoS_2_. Fig. (g) depicts the magnified optically stitched image of figure (f) in 1mm x1mm area. THz time-domain spectroscopic measurements were performed using THz beam with a spot size of 3-4 mm on specific location [shown in Fig. S9 (a)].  THz time-domain spectroscopy was also carried out on the bare substrate (without any growth), as well as on the selected growth locations as shown in Fig. S9(h). Fig. S9 (i), represent the magnified image of Fig. S9 (h) which is clearly demonstrating the role of the synthesized monolayer MoS_2_. It is evident from Fig. S9 (i) the THz signals (red and pink plot) of the synthesized sample differ substantially from the bare substrate (black plot). In this way, the intrinsic/ true response of monolayer MoS_2_ has been captured, which is devoid of any contribution from the substrate.

**Supporting References**

1. Papanai, G.S., et al. New insight into the growth of monolayer MoS_2_ flakes using an indigenously developed CVD setup: a study on shape evolution and spectroscopy. Materials Chemistry Frontiers **5**,5429-5441 (2021).

2. Tummala, P., et al., Application-Oriented Growth of a Molybdenum Disulfide (MoS_2_) Single Layer by Means of Parametrically Optimized Chemical Vapor Deposition. Materials. **13**: p. 2786 (2020).

3. Singh, A., et al., NaCl-assisted substrate dependent 2D planar nucleated growth of MoS_2_. Applied Surface Science. **538**: p. 148201 (2021).

4. Han, T., et al., Probing the growth improvement of large-size high quality monolayer MoS_2_ by APCVD. **9**: p. 433(2019).

5. Li, H., X.H. Zhang, and Z.K. Tang. Terahertz dielectric response and optical conductivity of layered MoS_2_. in The 9^th^ International Symposium on Ultrafast Phenomena and Terahertz Waves. Changsha, Hunan: Optica Publishing Group. p. TuK47 (2018).

6. Zhang, X., et al., Flux method growth of bulk MoS_2_ single crystals and their application as a saturable absorber. CrystEngComm. **17**: p. 4026-4032(2015).

7. Papanai, G., et al., Qualitative Analysis of Mechanically Exfoliated MoS_2_ Nanosheets Using Spectroscopic Probes. The Journal of Physical Chemistry C **123**, 27264-27271 (2019).
